# Some Facts and Phases of Syphilis That Interest the Dentist

**Published:** 1904-02-15

**Authors:** Don M. Graham


THE
### DENTAL REGISTER.

Vol. LVIII. FEBRUARY 15, 1904. No. 2.

### SOME FACTS AND PHASES OF SYPHILIS THAT INTEREST THE DENTIST.

BY DON M. GRAHAM, M.D., D.D.S.

Read before the Detroit Dental Society, January 14, 1904.

In discussing with our secretary, a few months ago, topics for the society's program, we concluded that syphilis was of sufficient importance to the dentist to demand an evening's consideration and discussion. I suggested that not only the presentation of a paper, but also subjects illustrating the different phases of the disease would be highly interesting and instructive. A few days later, to my surprise, I received a program announcing among other things, that I was to present a paper as well as to provide a clinic on the subject. While the paper is but a digest of what has already been written on this important subject, some attempt has been made to present such phases and facts as would appear to be of special interest to the dental practitioner. The writer has now learned what most of you already know, that human nature is not prone to parade its misfortune before the public. He therefore fears that his paper will not be profusely illustrated by living pictures. However, the evening will be profitably spent, I doubt not, in the discussion which will follow.

Were I to say that the average dentist in Detroit recognizes not more than one of two cases of syphilis in the course of a year, I would feel that the members of this society would agree with me, while the reply from our medical brethren, I fear, would be "because you are unable to recognize it." So eminent an authority as Dr. Osler says, "there are probably more families with a luetic than a tubercular taint." Statistics are incomplete, but probably not less than 2 percent of the inhabitants of civilized countries are suffering from one form or another of this disease, while in some semi-civilized countries as high as 25 and 30 percent are sufferers, and of this large number of cases, we are told that not less than 25 percent are contracted innocently. This is enough in itself to commend the subject to our thoughtful consideration that we may become equipped in the best possible manner to prevent the spread of this dread disease.

Syphilis may assume the inherited or acquired form. The manifestations of the acquired form are evident in the new-born, which is poorly nourished, emaciated, and often with blisters on wrist and ankles. If the infant be born plump and healthy, the inherited taint shows itself usually within four to eight weeks by a coryza or "snuffles," and a faulty nutrition. The lips and angles of the mouth are fissured and ulcerated. The secretions from these lesions, being very virulent, are the source of infection to the wet nurse and others, while the mother enjoys perfect immunity while nursing and handling her child. If the child lives to the age when his permanent upper centrals erupt, we generally find the typical "screw-driver" or Hutchinson teeth; these are somewhat rounded and peg-shaped, tapering from gum to cutting edge. This type of tooth is not an infallible evidence of congenital syphilis, but it is said that if with this we have keratitis and middle-ear disease, a positive diagnosis of hereditary syphilis may be made.

For the sake of convenience, syphilis is divided into three stages—primary, secondary and tertiary. The primary lesion usually begins as a red papulæ which, after a few weeks, slowly ulcerates in the center forming an indurated base of gristly cartilaginous consistence, hence the name hard or indurated chancre. The chancre is usually single and may partake of the nature of the abrasion where infection has taken place, thus a fissure, ulcer, or vegetation may somewhat alter the form of the chancre. Accompanying chancres we usually have glandular enlargements; with genital chancres we have inguinal enlargements; with labial or lingual chancres we have submaxillary or sublingual enlargements which follow in a few days after the appearance of the chancre. They differ from the infectious enlargements in that they are indurated and freely movable, and are neither painful nor inflammatory unless secondary infection ensues.

In from six to twelve weeks constitutional symptoms appear which mark the beginning of the secondary stage. These may appear insidiously or suddenly, as after a hot bath, alcoholic stimulation or violent exercise, as cutaneous eruptions. It is with the secondary stage that the dentist is mostly concerned; and of all the symptoms, the mucous patches are the most characteristic and diagnostic, and the most evident to the dental practitioner. In one important point syphilitic lesions differ from other cutaneous lesions in that they are seldom attended by subjective symptoms although often extensive. They are rarely accompanied by local inflammations, although fever and anemia are frequently features of the disease. The contour of syphilides is often suggestive. In single lesions or in groups of aggregate lesions, whether of mucous patches or cutaneous eruptions, we often see the circular conformation. Mucous patches, ulcers and scars often observe the curve or segment of a circle, or assume a serpiginous aspect. The mouth lesions are but manifestations of the explosion which is elsewhere expressed in skin eruptions, as cutaneous lesions are often difficult of diagnosis, so also are these epithelial lesions.

It is the syphilitic behavior rather than the syphilitic lesion that guides the diagnostician, and the dentist should confirm or allay suspicion by a careful consideration of all the evidence that can be brought to bear on the case. This embarrassment and delicacy frequently can be avoided by communicating with the family physician, who should be ever willing to co-operate with the dentist in this matter, and who owes it to the dental fraternity, and the community to convey such evidence to the family dentist, that he may be able to prevent infection. On the other hand, if the dentist be the first to suspect syphilis he should refer the patient to a competent physician instead of treating mouth lesions, as I have known some dentists to do.

Beginning with an explosion of general or secondary syphilis, we have a pharyngeal or nasal blush often extending to the tonsils, and interfering with swallowing, occasioning sometimes a hoarseness and cough. These patches form on the mucous membrane and indeed on all moist surfaces of the body. They are but papuels moistened, macerated and flattened, by juxtaposition of neighboring tissues. These roundish or oval lesions are flattened or slightly depressed, pale-rose or whitish spots, and although they are generally characteristic and diagnostic of syphilis, they should not in themselves warrant a positive and conclusive diagnosis. They should be carefully differentiated from simple aphthous patches, caused by indigestion, and local disturbances, such as smoking or macerations from biting, etc. In external features these patches may very closely resemble each other, but only in syphilis have we systemic infection. Of simple aphthous sores, we have for the most part singleness and generally exquisite tenderness, with mucous patches, multiplicity is the rule, and much less soreness, although with secondary infection soreness may be a prominent symptom. Cankers, linear streaks and bands are often found on the inner cheeks representing contact of upper and lower teeth on these tisues, while behind the lower third molar representing contact by upper third molar we sometimes find a lucoplastic area, but careful examination will always reveal the true nature of such lesions.

The tertiary stage, consisting of gummata, visceral syphilis and sequele is not of so much interest to the dentist since the danger of infection is almost *nil.* He is very rarely called upon to replace tissue which has suffered necrosis since early and proper medical treatment is sufficient to prevent such a catastrophe. In neglected or unfortunate cases, however, a vulcanite plate gives much satisfaction and comfort to the unfortunate as in the case of a perforate palate.

In concluding this paper, which is primarily of dental interest I would suggest that we remember the prevalence of this disease and its awful consequences. While all instruments coming in contact with the tissues of the mouth should be thermally sterilized, at the end of the day's work, especial precautions should be taken where syphilis is suspected. Hands should be disinfected and instruments should be boiled for at least twenty minutes in a solution of carbonate of soda, which serves the double purpose of dissolving fats and preventing instruments from rusting. It has been advocated, and I think wisely, that two sets of instruments should be kept, the one employed exclusively in infected and suspected cases. The extra set does not necessarily entail much expenditure as only temporary work need be attempted during the few months the syphilitic lesions are most numerous and the virus most virulent. A few burs, scalers, and excavators are all that is necessary. With modern scientific methods of treating this disease, the teeth of the syphilitic should require no more treatment than the non-syphilitic, although all broken down teeth should be extracted, and necessary fillings inserted with some of the plastic or easily-inserted materials.

